# You don’t win friends with bad salad! A gene editing approach to enhance the powdery mildew resistance in cucumber

**DOI:** 10.1093/plphys/kiae160

**Published:** 2024-03-14

**Authors:** Sara Selma

**Affiliations:** Assistant Features Editor, Plant Physiology, American Society of Plant Biologists; VIB Center for Plant Systems Biology, Ghent 9052, Belgium

One of the biggest challenges for agriculture lies in finding strategies that minimize crop yield loss due to pests and diseases. Powdery mildew (PM) is a widespread fungal disease that affects a diverse range of crops; for example, in cucumber (*Cucumis sativus L.*), PM can cause losses of up to 40% (He et al. 2022). Various studies have focused on identifying PM resistance (PMR) genes that benefit cucumber breeding programs (Liu et al. 2008). The characterization of quantitative trait loci (QTL) for mapping PMR linked the disruption of the *Mildew resistance Locus 8* (*CsMLO8*) gene with the PM resistance in cucumber. However, although the loss of function of *CsMLO8* is indispensable for the PMR, it is not enough to generate a complete resistance (Nie et al. 2015a, 2015b; Berg et al. 2015). The PM-resistant QTL also contains other members of the *CsMLO* family, pointing out that more than 1 *CsMLO* gene is involved in PM resistance (Schouten et al. 2014). A recent study characterized the MLO proteins as calmodulin-gated calcium channel proteins (Gao et al. 2022), suggesting that calcium signaling is involved in *mlo*-mediated PM resistance. However, both the components and mechanism of PM resistance remain not fully understood.

In this issue of *Plant Physiology*, Ma et al. 2024 identify the *CsMLO* genes involved in the resistance against PM infection. In addition, they validated the link between the *Csmlo-*mediated PM resistance and the calcium signaling pathway through the identification of novel calcium-related proteins in the PM resistance.

**Figure 1.**
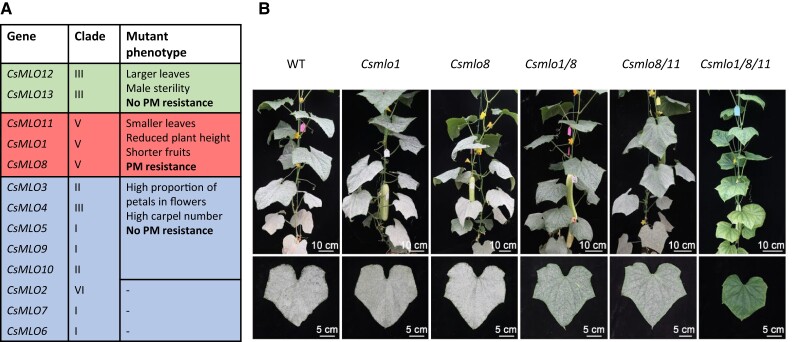
Identification of the *CsMLO* genes involved in the resistance against PM infection. **A)** Clade classification of the *CsMLO* genes accompanied the phenotypic observation of the *Csmlo*-mutated plants compared to WT. The green-colored box represents the genes edited with 2-target construct. The red-colored box represents the genes edited with 3-target construct. The blue-colored box represents the genes edited with 8-target construct. No loss-of-function mutation was obtained from the *CsMLO2*, C*sMLO7*, and *CsMLO6* genes. **B)** PM resistance phenotypes of WT and clade V *Csmlo* mutants. The grayish-white powder on the leaves indicates the presence of the fungus. The phenotypes were documented at 20 days post infection (dpi) in the greenhouse. Figure extractect from Ma et al. 2024.

As a starting point, the authors evaluated the potential role of the *CsMLO* family in PM resistance by mutating all the *CsMLO* members using CRISPR/Cas9-based multiplex gene editing (Čermák et al. 2017). The CRISPR-Cas9 system is a widespread method for genome editing that consists of a Cas9 protein, which acts as a pair of “molecular scissors” of the target DNA and a small piece of RNA (gRNA) that drives the Cas9 to the target DNA sequence. The *CsMLO* family contains *13 CsMLO* genes divided into 5 different clades (Fig. 1A), so the authors optimized the CRISPR-Cas9 system to perform an efficient multiplexing approach in cucumber that allows the expression of several gRNAs at the same time. Eight-target, 3-target, and 2-target constructs were designed to mutate the 13 *CsMLO* members (Fig. 1A), obtaining 17 independent T0 transgenic plants with high editing efficiency. *Csmlo* mutants were generated in less than 2 generations, highlighting the success of the multiplexed gene editing system developed by the authors.

The growth phenotypes of *Csmlo* mutants were assessed to point out the function of the *CsMLO* clades but also potential pleiotropic effects that might alter the yield and quality of the crops. The results show that the 8-target construct employed to assess clades I, II, III, and VI resulted in the frameshift mutations of *Csmlo5*, *Csmlo3*, *Csmlo3/10*, *Csmlo3/5/10*, *Csmlo3/4/5/10*, and *Csmlo3/4/5/9/10*. Notably, flowers from these mutants showed an increased proportion of petals and higher carpel numbers compared to the wild-type (WT). On the other hand, the *Csmlo12/13* mutant exhibited larger leaves than the WT and male sterility due to hindered pollen germination. Finally, the *Csmlo1/8/11* triple mutants (clade V) presented smaller leaves, reduced plant height, and shorter fruits compared to the WT. The results highlight the significant role of the *CsMLO* family in diverse developmental stages.

Regarding PM resistance in the *Csmlo* plants, the results show that the *Csmlo8* single mutant was fully susceptible to PM, with no significant difference compared to the WT. Interestingly, the double mutants *Csmlo1/8* and *Csmlo8/11* showed moderate resistance, and the triple *Csmlo1/8/11* exhibited complete PM resistance (Fig. 1B). These results support the previous studies that concluded that *CsMLO8* disruption was insufficient but necessary to generate PM resistance, demostrating a functional redundancy among clade V. The rest of the *CsMLO* loss-of-function lines were equally susceptible than the WT to PM, indicating that this function is restricted to the *CsMLO* clade V in cucumber.

Additionally, the authors performed a transcriptomic and proteomic analysis to unravel the molecular mechanisms underlying PM resistance using the susceptible WT, *Csmlo1/8* with moderate PM resistance, and *Csmlo1/8/11* with complete PM resistance. The differential expression analysis comparing the PM-susceptible and PM-resistant genotypes matches with the proteomics results revealing a functional enrichment of genes involved in “plant-pathogen interaction” and “calcium ion binding.” The candidates KCBP-interacting Ca^2+^ binding protein (CsKIC), CaM-like protein 28 (CsCML28), and Ca^2+ −^dependent protein kinase 11 (CsCPK1) were selected from the enriched “calcium ion binding” category as potential new players involved in the PM resistance (Kim et al. 2002; Freymark et al. 2007). CsKIC exhibited both mRNA and protein upregulation in *Csmlo1/8/11*, while *CsCML28* and *CsCPK11* showed drastic protein changes without significant mRNA alterations, suggesting a possible post-transcriptional and post-translational regulation. Yeast-2-hybrid assays reveal that CsKIC can interact with the C terminus of CsMLO8 but also with the C termini of CsMLO1 and CsMLO11, indicating possible collaborative functions. No interactions were observed between CsKIC, CsCML28, and CsCPK11.

To further investigate the role of *CsKIC*, *CsCML28*, and *CsCPK11* in cucumber’s resistance to PM, a virus-induced gene silencing approach was employed to downregulate their expression. Silencing *CsKIC* and co-silencing of *CsKIC* with *CsMLO8* resulted in enhanced PM resistance, indicating that *CsKIC* acts with *CsMLO8* as a negative regulator of PM resistance. On the contrary, silencing *CsCML28* and *CsCPK11* increased susceptibility to PM, indicating that they act as positive regulators in PM resistance.

In summary, the work by Ma et al. 2024 uncovered genes involved in cucumber PM resistance. Although the triple mutant *Csmlo1/8/11* is completely resistant to the PM infection, it shows significant growth penalties not desired in the breeding programs. In addition, the role of the Ca^2+^ binding proteins *CsKIC, CsCML28*, and *CsCPK11* in the *Csmlo*-mediated PM resistance could offer new strategies to obtain PM-resistant varieties. However, this calcium-dependent mechanism is still not completely understood. Future studies may focus on that direction to benefit not only cucumber breeding programs but other PM-sensitive crops.
